# PK-profiling method for identifying the expression of resistance-associated genes in partially resistant oats to crown rust

**DOI:** 10.1186/s12870-018-1604-y

**Published:** 2018-12-29

**Authors:** Yolanda Loarce, Pilar Dongil, Araceli Fominaya, Juan M. González, Esther Ferrer

**Affiliations:** Department of Biomedicine and Biotechnology, University of Alcalá, Campus Universitario, 28805 Alcalá de Henares, Madrid Spain

**Keywords:** Partial resistance, PK-profiling, Subtraction library, Protein kinases

## Abstract

**Background:**

Protein kinases play a key role in plant cell homeostasis and the activation of defense mechanisms. Partial resistance to fungi in plants is interesting because of its durability. However, the variable number of minor loci associated with this type of resistance hampers the reliable identification of the full range of genes involved. The present work reports the technique of protein kinase (PK)-profiling for the identification of the PK genes induced in the partially resistant oats line MN841801–1 following exposure to the fungus *Puccinia coronata*. This is the first time this technique has been used with cDNA (complementary DNA) from a suppression subtractive hybridization library obtained after the hybridization of cDNAs from inoculated and mock-inoculated plants.

**Results:**

Six degenerate primers based on the conserved domains of protein kinases were used in a PK-profiling assay including cDNA from mock-inoculated leaves and subtracted cDNA. Of the 75.7% of sequences cloned and sequenced that showed significant similarity to resistance genes, 76% were found to code for PKs. Translation and ClustalW2 alignment of each sequence cloned with the complete sequences of the most similar *B. distachyon* PKs allowed those of the partially resistant oat line to be deduced and characterized. Further, a phylogenetic study carried out after alignment of these *B. distachyon* PK sequences with the most similar protein sequences of related species also allowed to deduce different functions for the PK cloned. RT-qPCR (Reverse Transcription-quantitative PCR) was analyzed on nine representative sequences to validate the reliability of the employed PK-profiling method as a tool for identifying the expression of resistance-associated genes.

**Conclusions:**

PK-profiling would appear to be a useful tool for the identification of the PKs expressed in oats after challenge by *P. coronata*, and perhaps other pathogens. Most of the PKs studied are related to receptor-like protein kinases expressed shortly after infection. This is in agreement with previous studies indicating a close relationship between partial resistance and the first layer of defense against pathogen used by plants.

**Electronic supplementary material:**

The online version of this article (10.1186/s12870-018-1604-y) contains supplementary material, which is available to authorized users.

## Background

Despite decades of effort invested in deciphering the mechanisms involved in plant-pathogen interactions, and in the characterization of the genes with a role in defense responses [1, 2], the annual crop losses caused by biotrophic agents highlight the need to continue the search for new resistance genes and to fully understand plant defense mechanisms.

Plant qualitative or race-specific defense is characterized by complete resistance following the recognition, by the products of the plant’s *R* genes (Resintance genes), of the products derived from the pathogen’s *Avr* genes (Avirulence genes). Such recognition activates signaling cascades that initiate the expression of the defense genes. However, this type of resistance is unstable and short-lived. Mutations in *Avr* genes occur frequently, and a plant’s resistance might then be overcome.

Partial resistance is a type of incomplete resistance, frequently under polygenic control. In different plant species this form of resistance has been selected when monogenically mediated resistance is absent [3]. Partial resistance commonly achieves a reduction in pathogen multiplication and symptom severity. Although the pathogen survives, any evolutionary towards full virulence will be slowed [4].

Extensive research into host resistance in different pathosystems has revealed many components of the complex molecular mechanisms that operate in active plant defense [5]. The primary immune system of the plant is based on the synthesis of pathogen recognition proteins that trigger different signaling cascades in which protein kinases (PKs) play a key role [6]. The abundance of PK genes in plant genomes highlights their importance [7]. The plant genes coding for pathogen-recognizing proteins, of which there are different families, are also abundant*.* One family of recognition proteins with PK activity - the receptor-like kinases (RLKs) - plays an important role in the initial signaling of pathogen recognition, and in the subsequent activation of the first layer of defense mechanisms or basal defense [5, 8, 9]. RLKs are transmembrane proteins with a highly variable extracellular region, a single membrane-spanning domain, and an intracellular kinase domain - frequently a serine/threonine kinase domain, and more rarely a cysteine-rich kinase domain. The N-terminal domain defines the specificity of the ligand and divides plant RLKs into subfamilies. The most numerous subfamilies have leucine-rich repeats (LRR) [10]. However, cytoplasmic PKs also exist with only serine/threonine kinase domains, i.e., they have no transmembrane or LRR domains [6].

The second layer of plant innate immunity involves resistance proteins encoded by *R* genes; these recognize specific pathogen elicitors and unleash a robust defense reaction in which PKs are also involved. Several proteins coded by *R* cloned genes - show kinase domains what are able to initiate the defense response [11]. Moreover, different PKs participate as downstream active elements in plant defense signalling in both primary immunity and resistance mediated by R genes, like mitogen-activated protein kinases (MAPKs) and calcium-dependent protein kinases. Conserved domains have been described in a variety of receptor proteins and *R* gene products isolated from different plants. This feature has been widely exploited to identify similar sequences in other plant species using PCR. These sequences are generically known as resistance gene analogs (RGAs) ( [12–15] and references cited therein). When primers targeting conserved domain sequences - such as the NBS (Nucleotide Binding Site) domain of several *R* genes - are used in combination with polyacrylamide gels, the number of RGAs detectable can be increased. This technique, known as NBS-profiling, produces targeted markers. Information about the conserved domains of PK genes have also been used to design primers, allowing PK-profiling. Both NBS-profiling and PK-profiling generate reproducible, polymorphic, multi-locus banding patterns that have been successfully used to identify and map RGAs in different plant species [16–22].

Despite the abundance of RGAs in plant genomes ([23] and references therein), comparisons of their sequences with those deposited in expressed sequence databases revealed only a few to actually be expressed [24]. This hinders the use of genomic motif-directed profiling for the isolation of candidate *R* genes. Transcriptome sequencing of plants with different pathosystems has notably increased the number of functional RGAs known [25, 26], suggesting that the isolation of RGAs from cDNA might be a good tool for identifying functional candidate *R* genes.

Oats is an important food and animal feed crop. Unfortunately, the genomic resources available for oats are much reduced compared to those of other crops. Putative pathogen receptor and *R* genes from either genomic or transcriptome sequences have therefore been difficult to isolate. However, efforts have been made to characterize oat RGAs that might be useful in the development of genetic markers of resistance. Using individual RGA amplicons and motif-directed-profiling, a number of markers have been located in several oat genetic reference maps [14, 21]. Some of these markers lie close to previously mapped Mendelian genes for resistance. Similarly, a number of these markers have been located close to resistance QTLs (Quantitative Trait Loci) in the genetic map obtained from the cross of the oats line MN841801–1 (which shows partial resistance to the fungus *Puccinia coronata*) with the susceptible cultivar Noble-2 [4, 27]. However, the paucity of markers in this map prevents any stronger relationship being established among profiling markers and QTLs.

A molecular method for revealing the genes involved in partial resistance has been developed in oats. Recently, our laboratory analyzed the transcriptome of a suppression subtractive hybridization (SSH) cDNA library of MN-841801-1 after inoculation with *P. coronata* [28], and observed differences in the expression of genes between inoculated and mock-inoculated plants. Fifteen percent of the 929 genes identified had clear roles in defense and signal transduction.

Using cDNA from mock-inoculated plants of MN841801–1, along with cDNA from the above SSH library, PK-profiling was used to determine the PKs specifically induced in the partially resistant oats line MN841801–1 when under attack from *P. coronata*.

## Methods

### Plant material, pathogen, and inoculations

The plant material examined in this work was the MN841801–1 line of hexaploid *Avena sativa. L.* (provided by Dr. H. W. Rines of the Department of Agronomy and Plant Genetics, University of Minnesota, St. Paul, MN, USA). This line has shown partial resistance to different isolates of *P. coronata* for over 30 years. Seeds were grown in a growth chamber under a 14 h light (at 19 °C)/10 h dark (at 18 °C) cycle at 65% humidity, until the five leaf stage.

The 93MN8236 isolate of *P. coronata* Corda *f. sp. avenae* Erikss, provided by G. E. Ochocki of the Cereal Disease Laboratory, Agriculture Research Service, USDA (St. Paul, Minnesota, USA), was used to infect the plants. Inoculations were carried out as described by [28].

### cDNA PK-profiling

Total RNA was extracted from MN841801–1 leaves using Tripure Reagent (Roche, Germany) according to the manufacturer’s instructions, and treated with Turbo RNase-free DNase (Ambion, Thermo Fisher Scientific, Whaltman, MA, USA) to remove all contaminating DNA. Poly-A^+^ RNA was then extracted from the total RNA using The Dynabeads Oligo (dT)_25_ Kit (Invitrogen, Thermo Fisher Scientific, Whaltman, MA, USA). cDNA was synthesized from 40 ng Poly-A^+^ RNA employing the SMARTer PCR cDNA Synthesis Kit (Clontech, Takara Bio, Shiga, Japan) according to the manufacturer’s instructions.

PK profiling from cDNA of mock-inoculated plants was performed as reported in van der Linden et al. [16] with the modifications described by [21]. The restriction enzyme *Rsa*I was used to digest 100 ng of cDNA. Adapters were then ligated to the restriction sites. The digestion of the cDNA and ligation of the adapters was performed in a single reaction at 37 °C. PK-specific fragments were PCR-amplified using primers directed towards specific sequences encoding amino acid motifs in conserved PK domains. The primers used were PK1Fa, PK1Fb, PK3Fa, PK3Fb, PK4R1a and PK4R1b, previously described by [20]. These were used in combination with adapter primers also described in [20]. The annealing temperature was set at 55 °C. The amplicons were then reamplified using the same adapter primers but labeled with the fluorochrome IRDye700 to visualize individual fragments in 6% denaturating polyacrylamide gels using the LI-COR 4300 DNA Analysis System (LI-COR Biosciences, Lincoln, NE, USA).

cDNA from an SSH library, obtained by [28], was also subjected to PK profiling.

### Cloning and characterization of PK markers

For the cloning of PK polymorphic fragments, the PCR amplicons were fractionated by electrophoresis in a 15 cm-long 2% agarose gel. The fragments were excised and the DNA extracted using the QIAquick Gel Extraction Kit (QIAGEN Inc., Venlo, Netherlands). DNA was cloned into the pGEM-T vector (Promega, Madison, WI, USA). Five clones per fragment were then PCR-amplified using vector primers, and three clones randomly selected for sequencing. All sequencing reactions were performed at the Molecular Biology Unit of the University of Alcalá (Alcalá de Henares, Spain). Sequence analysis was performed using software CodonCode Aligner v.3.7.1 (CodonCode Corporation, Centerville, MA, USA). Finally, sequences were compared with those in the NCBI database using BLASTn and BLASTx software [29]. The small length of sequences obtained in the PK profiling preclude a fully characterization of the putative PK isolated. To have a more complete information on the nature of cloned oat sequences, *B. distachyon* protein sequences with the highest similarity with each oat PK cloned were used. Among the Poaceae, *B.distachyon* has shown the closest relationship with *Avena* species [30]. These putative *B. distachyon* orthologous sequences were aligned with the oat PK derived from the PK-profiling using ClustalW2 software at EMBL-EBI. PROSITE software [31] was used for the identification of conserved PK motifs.

To better understand the role and function of cloned oat PK sequences, their putative orthologous or paralogous full-length sequences from species close related to *Avena*, like *B. distachyon*, *Triticum tausch*i, *T. aestivum*, *Hordeum vulgare* and *Oryza sp* were retrieve from NCBI and aligned. A phylogenetic analysis was carried out with the Neighbor-Joining method of the Simple Phylogeny at EMBL-EBI. The phylogenetic tree was visualized with the Tree Viewer of ETE Tool kit [32].

### RT-qPCR

The relative quantification of gene expression was performed for a selected number of sequences (identified by PK-profiling) that showed similarity to PKs (Additional file 1). Leaf tissues inoculated with *P. coronata* were sampled at 24, 48 and 72 h post-inoculation (hpi) along with those of control plants -mock-inoculated with sterile water- sampled at the same point times that the infected plants (experiments were performed in triplicate). cDNA from the mock-inoculated and pathogen-inoculated leaves were prepared as above described and RT-qPCR reactions were conducted following [28]. All samples were run in duplicate in a 7500 Fast Real Time PCR System Thermocycler (Thermo-Fisher Scientific, Whaltman, MA, USA). Ct (threshold point) values were determined and the relative expression level for each gene in the different templates calculated using the qBase Plus method [33], normalizing against the genes coding for malate dehydrogenase (MDH) and CDC48 ATP-ase. At each sample point, the difference in relative expression was deemed significant when the expression in the pathogen-inoculated plants was twice that seen in the mock-inoculated plants.

## Results and discussion

### PK-profiling

**Fig. 1:**
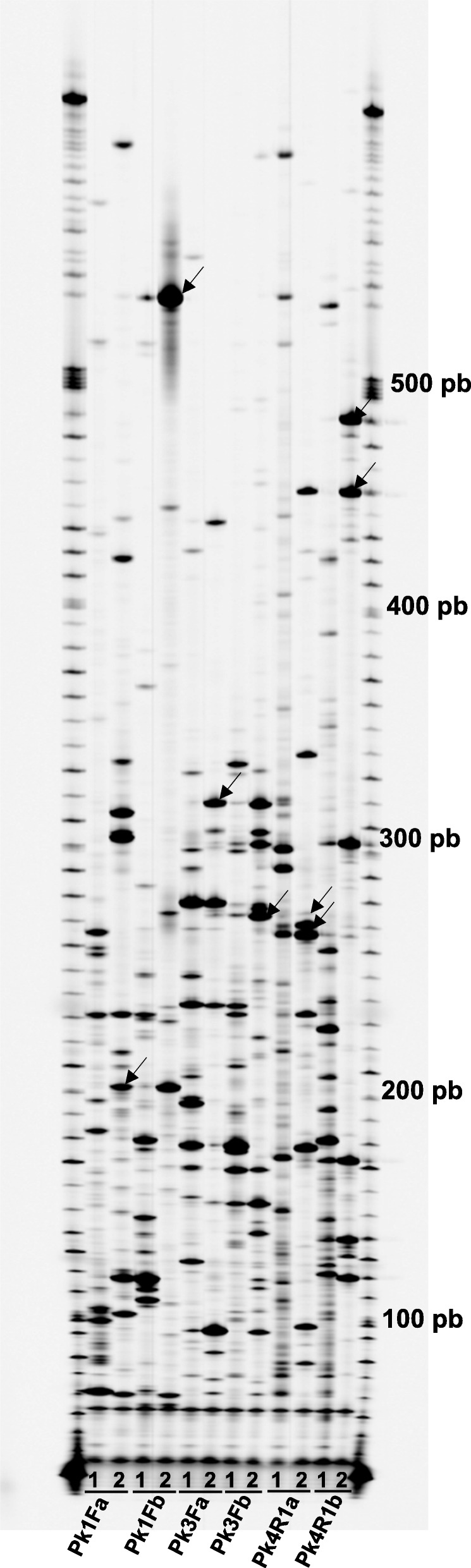
PK-profilings of cDNA from the mock-inoculated resistant line used as control (lanes 1), and cDNA from MN841801–1 coming from the SSH library (lanes 2) with the set of PK primers used. First and last rows correspond to weight molecular markers, reference sizes are indicated on the right. Arrows indicate excised PK fragments analyzed by RT-qPCR

**Fig. 2:**
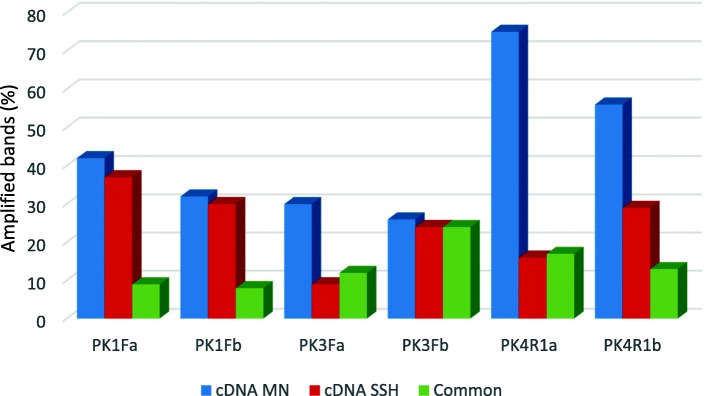
Comparison of the number of bands obtained in the PK-profiling with the set of PK primers. cDNA MN stands for specific fragments from mock inoculated cDNA from MN841801–1; cDNA SSH stands for specific fragments from the cDNA of the SSH library; common stands for fragments found in both types of cDNA

The sequences of the 6 degenerate primers used in PK-profiling were based on the conserved domains of PKs [20]. These primers, and the enzyme *Rsa*I, were chosen since they were shown to be the most efficient in previous experiments [21]. Patterns of bands ranging between 50 and 700 bp, showing different intensities, were generated (Fig. 1). Significant differences were observed in the number of bands generated by each primer (between 51 and 108 bands). PK4R1a and PK4R1b produced the most bands (108 and 98 respectively), while PK3Fa, produced the fewest (51). Figure 2 shows the number of bands amplified by each primer pair. The range of bands is similar to that described in other studies involving motif-profiling with cDNA as a template [22, 34]. Naturally, the band profile was much less complex than that obtained by other authors who used genomic DNA from different plant species as a starting material [16, 18, 20]. The reduced number of bands implies the isolation of a potentially smaller number of specifically targeted sequences and, therefore, of putative PK gene family members. However, since they all represent expressed genes, the chances of obtaining functional sequences are higher [22].

A grand total of 489 bands were amplified (Fig. 2). As expected, because of the subtraction process, the number of total specific fragments was clearly larger for the cDNA from the mock-inoculated leaves than from the subtracted cDNA (261 vs. 145). The subtracted DNA ought therefore to be enriched in sequences specifically expressed in response to the pathogen and with a poorer representation of constitutive expressed sequences. Since the publication of [35], the SSH technique has been employed in a wide range of studies. Its main purpose has been to obtain either cDNA probes or libraries of cDNA sequences differently expressed under different conditions or in different tissues. The technique has been successfully used to isolate plant genes expressed in different plant-pathogen interactions [28, 36, 37].

Eighty-three of the 489 bands (17% of the total number) were common between the two types of samples, therefore, they were taken as monomorphic. The existence of monomorphic bands does not necessarily mean they represent the same fragment; fragments of identical size can possess different nucleotide sequences. If they do represent the same fragment, their presence in the SSH cDNA might be explained as a failure in the subtraction process. Dougherty and Geschwind [38] indicate that some sequences may persist after mild subtraction. Hence, the fewer the number of subtraction rounds, the larger the number of common sequences left - and in the present work there was only one round of subtraction. Thus, part of these monomorphic sequences might correspond to constitutive unsubtracted sequences. However, the PCR technique involved might increase the ‘visibility’ of any differences in later gel electrophoresis.

Comparison of the mock-inoculated and SSH cDNA PK-profiles revealed 406 specific bands (83%). As a whole, 261 were fragments unique to the control cDNA representing a 53.37% of the number of total bands, whereas 145 (29.65%) were specific fragments to the PK-profile from the subtracted cDNA. Numbers of bands (monomorphic, unique to mock-inoculated DNA or unique to subtracted cDNA) analyzed with each primer are also shown in Fig. 2. Microarrays and RNA-seq (RNA sequencing) experiments have amply documented the changes in the transcriptome that occur because of pathogen infection, reviewed in [39, 40]. Thus, genes expressed in control conditions, but not during infection, might have been downregulated in response to pathogen attack and should be thus absent from the subtracted PK-profile. Conversely, the fragments specific to the subtracted cDNA might be assigned to sequences upregulated consequently to the infection. Taken together, these results indicate that SSH is an effective means of identifying genes predominantly expressed by the MN84810–1 plants when infected.

### Cloning of specific SSH PK-profiling bands

To overcome the difficulties of handling the polyacrylamide gels and the visualization of bands in a UV transiluminator, highly concentrated, 15 cm-long agarose gels were used. Comparisons of the control and subtracted cDNA profiles allowed specific bands of subtracted cDNA to be selected for cloning. As the size of the amplified bands in the PK-profiling assays is small, only the bands large enough to provide information were selected. A total of 17 bands were cut out of the gel and named according to degenerate PK primer used in their amplification, followed by a number indicating the relative position of the polymorphic band in the gel. At least five clones from each fragment were reamplified by PCR to determine the size of the insert. In total, inserts of 115 clones were amplified, and 63 were sequenced. Twenty-eight different sequences were obtained. Table 1 shows clones with non-redundant sequences obtained with each PK primer (GenBank accession numbers JZ978700-JZ978726). Occasionally, different primers produced fragments with the same sequence, for example, PK3Fa14 and PK3Fb11. The use of several degenerated primers targeting a same motif with the mild conditions of the PCR annealing temperature would contribute to the amplification of a same cDNA sequence in different PCR reactions.

**Table 1:** Most representative clones obtained with each PK primer

Primer	Clones (accession no)	Size (bp)	BLASTx function (*Brachypodium* accession numbers)
PK1Fa	PK1Fa114 (JZ978700)	180	Multidrug and toxin extrusion transporter protein (MATE) (XP_010233738)
	PK1Fa113 (JZ978701)	159	Peptidase protein (XP_00357982)
	PK1Fa115 (JZ978702)	150	LRR receptor-Mixed protein kinase (XP_014758670)
	PK1Fa123 (JZ978703)	67	Hypersensitive response protein (XP_003557236)
	PK1Fa2312 (JZ978704)	250	Receptor-like serine/threonine-protein *kinase (XP_003569123)*
	PK1Fa2313 (JZ978705)	137	No significant similarity found
	PK1Fa2315 (JZ978706)	159	Peptidase protein (XP_003579821)
	PK1Fa2321 (JZ978707)	251	Receptor-like serine/threonine-protein *kinase (XP_003569123)*
	PK1Fa2324 (JZ978708)	261	ADP-ribosylation factor (XP_003570479)
PK1Fb	PK1Fb111 (JZ978709)	478	Cysteine-rich receptor-like protein Mixed kinase (XP_003562979)
	PK1Fb113 (JZ978710)	256	Cysteine-rich receptor-like protein Mixed kinase (XP_003562978)
	PK1Fb212 (JZ978711)	343	Cysteine-rich receptor-like protein Mixed kinase XP_010237455)
	PK1Fb321 (JZ978712)	150	LRR receptor-Mixed protein kinase (XP_014758670)
PK3Fa	PK3Fa14 (JZ978713)	269	Pto-like interacting Mixed protein kinase (XP_003568986)
	PK3Fa23 (JZ978714)	227	Glyceraldehyde-3-phosphate dehydrogenase (GAPDH) (XP_003574540)
PK3Fb	PK3Fb11 (JZ978713)	269	Pto-like interacting Mixed protein kinase (XP_003568986)
	PK3Fb12 (JZ978715)	224	Calcium-dependent Serine/threonine protein kinase (XP_003580397)
	PK3Fb21 (JZ978714)	227	Glyceraldehyde-3-phosphate dehydrogenase (GAPDH) (XP_003574540)
	PK3Fb25 (JZ978716)	106	Serotonin N-acetyl protein transferase (XP_020178557.1)
PK4R1a	PK4R1a11 (JZ978717)	223	LRR receptor-like serine/threonine-protein kinase (XP_003563758)
	PK4R1a13 (JZ978718)	213	LRR receptor-like Mixed protein kinase (XP_003568447)
	PK4R1a14 (JZ978719)	220	LRR receptor-like Mixed protein kinase (XP_003568447)
	PK4R1a15 (JZ978720)	186	Chaperone protein (XP_003569728)
	PK4R1a24 (JZ978721)	132	SAGA factor (XP_003576694)
PK4R1b	PK4R1b11 (JZ978722)	256	Serine/threonine-protein kinase (XP_003566547)
	PK4R1b15 (JZ978723)	184	Ribulose-1,5-bisphosphate carboxylase/oxygenase (RuBisCO) (XP_003576920)
	PK4R1b21 (JZ978724)	430	Receptor serine/threonine kinase-like protein Xa21 (XP_010236334)
	PK4R1b22 (JZ978725)	400	LRR Mixed kinase SERK (XP_003577373)
	PK4R1b32 (JZ978726)	196	Alanyl-tRNA-synthetase (XP_003571715)

No redundant clones obtained with each PK primer, GenBank accession numbers, sizes of the cloned fragments and expected functions for the most similar protein sequences of *Brachypodium distachyon* accessions searched by BLASTx

BLAST searching of the NCBI databases revealed 75.7% of the sequences to show significant similarity to genes involved in defense against pathogens. 76% of these were classified as coding for PKs; they all had the typical serine/threonine kinases or serine/threonine-tyrosine domains (mixed kinases), and all showed similarity to kinases involved in defense responses (Table 1). InterProt searchs revealed that several also contained other features typical of the receptors identified as participants in defense processes, such as LRRs, cysteine-rich repeats (CRRs) or calcium binding sites. This strong similarity with sequences involved in defense has been reported by other authors using genomic DNA in NBS- and PK-profiling [16, 20]. In the latter experiments some 50–90% of the sequences obtained were similar to PKs.

The remaining 24% of the sequences showed similarity to molecules other than kinases but, interestingly, also involved in the plant defense response. For example, proteins involved in the activation of the hypersensitive response (PK1Fa123), and enzymes such as peptidases (PK1Fa113, and PK1Fa2315) that degrade fungal cell walls. Synthetases such as alanyl-ARNt synthetase (PK4R1b32) responsible for recognizing the amino acid alanine (an exogenous activator of abiotic resistance), and GTPases such as Arf (ADP-ribosylation factor) involved in postraductional modification (PK1Fa2324), essential in launching an effective defense [41]. The use of degenerate primers and the low stringency PCR conditions used might explain the presence of sequences not related to PKs. However, the fact that this 24% of all sequences could relate to defense mechanisms reveals the appropriateness of the subtraction procedure followed.

Finally, 24.3% of the total number of cloned fragments contained sequences not directly related to defense against pathogens, but responsible for growth and perhaps differently regulated under different circumstances. BLAST analysis revealed them to be similar to genes involved in metabolism and transport. Their sequences showed homology to PK sequences. For example, GA3P-DH (PK3Fa23 and PK3Fb21), which participates both in the glycolytic pathway and in the regulation of transcription and apoptosis. However, GAPDHs (GlycerAldehyde-3-Phosphate DeHydrogenases) may also play roles in abiotic stress tolerance [42]. Ribulose 1,5-diphosphate carboxylase (PK4R1b15), involved in photosynthesis and photorespiration but which also functions as a nitrogen storage protein and a potential source of N for the production of defensive metabolites in plants [43]. Serotonin N-acetyl transferase (PK3Fb25), involved in the transport of serotonin, a molecule that suppresses the growth of fungal hyphae in rice leaf tissue, reviewed in [44]. And certain proteins such as the ‘mate efflux’ (PKFa114) proton-dependent transporter [45], and the SAGA (Spt-Ada-Gcn5-Acetyl transferase) factors (PK4R1a24) which remodel chromatin to facilitate the access of the transcription machinery to it, favoring gene expression (Table 1). The results obtained indicate that most of the sequences isolated by PK-profiling were in fact taking part in the defense response implemented by these partially resistant oat plants.

### RT-qPCR

**Fig. 3:**
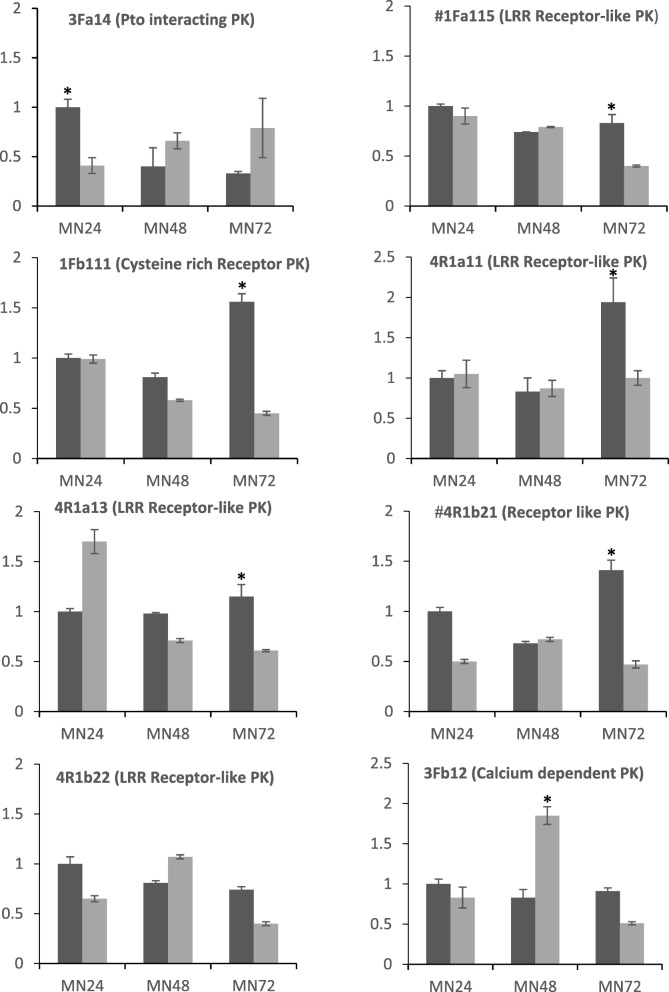
Time-course expression profiles of nine PK sequences in the partial resistant genotype MN841801–1 following inoculation with *P. coronata* (dark boxes) and after mock-inoculation (grey boxes). The relative expressions of genes are represented as the mean ± SE for the results of two independent RT-qPCR experiments. Within each diagram data are in relation to the expression level of the sample MN841801–1, 24 hpi after pathogen inoculation (relative expression =1). A gene was considered significantly induced or repressed when the ratio between the inoculated and control plants was > 2 or < 0.5, respectively. An asterisk stands up for significant differences. # Stands up for sequences analyzed in [28]

To validate the reliability of the present PK-profiling, RT-qPCR was performed on six representative sequences of the different categories of fragments only seen in the profiles of the SSH cDNA (Fig. 3). In addition, data from two other sequences indicated with an asterisk (1Fa115 and 4R1b21) obtained in these PK profiles were included. These also were analyzed in the sequencing process carried out on the SSH library [28] and were described under the names of isogroups 00320 and 00355. Differences in expression between pathogen-inoculated and mock-inoculated leaves were detected for 8 out 9 of the sequences. Seven out the nine sequences showed induced expression in the pathogen-inoculated plants. Differences were majority observed between pathogen-inoculated plants and mock-inoculated plants at 72 hpi. This agrees with the expression of other sequences from the SSH library previously studied [28]. This time point (72 hpi) might be an important time point for the induction of PKs involved in defense and should be used in further experiments analyzing the contrasting expression pathways of other resistant and susceptible oat genotypes. Only one of the analyzed sequences was downregulated at 48 hpi (3 Fb12). Another one (4R1b22) did not show expression differences at the time points scored. Therefore, the procedure to select and clone cDNA fragments specifically expressed in the pathogen-inoculated samples was fair efficient seven out of nine PK sequences showed upregulation in some of the time point scored. Moreover, results obtained reinforce the adequacy of the level of subtraction performed for the SSH library construction. This aimed to diminish downregulated and constitutive sequences in the cDNA from pathogen-inoculated plants and, conversely, to enrich cDNA samples in low abundance transcripts.

#### Characterization of the cloned protein kinases

**Fig. 4:**
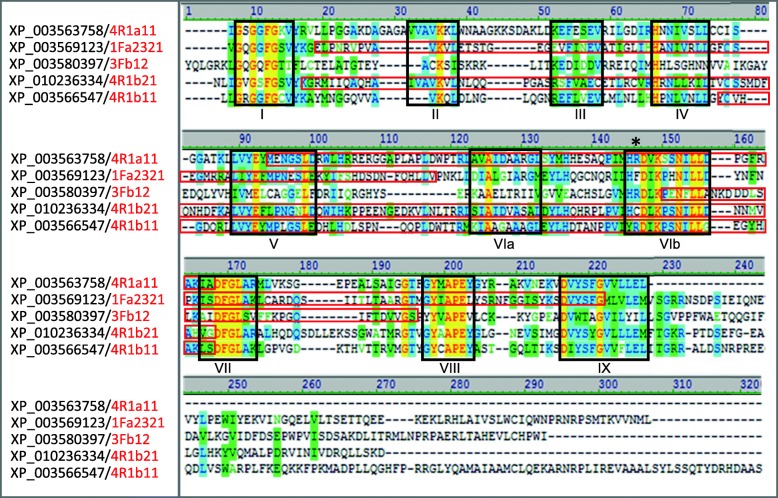
Alignment with ClustalW2 of the amino acid sequences of the serine/threonine kinases of *Brachypodium* (black) with the highest similarity to those obtained in the PK-profiling (red). Black boxes indicate the 12 conserved subdomains of the protein kinases, with the exception of the subdomain X that are poorly conserved. Red boxes show the sequences cloned in this work. Asterisk points to the characteristic sequence of the active center (HRD). The colors of the amino acid refer to the degree of similarity: Black: Not similar; Yellow: Identical; Green: Similar; Blue: Conserved

**Fig. 5:**
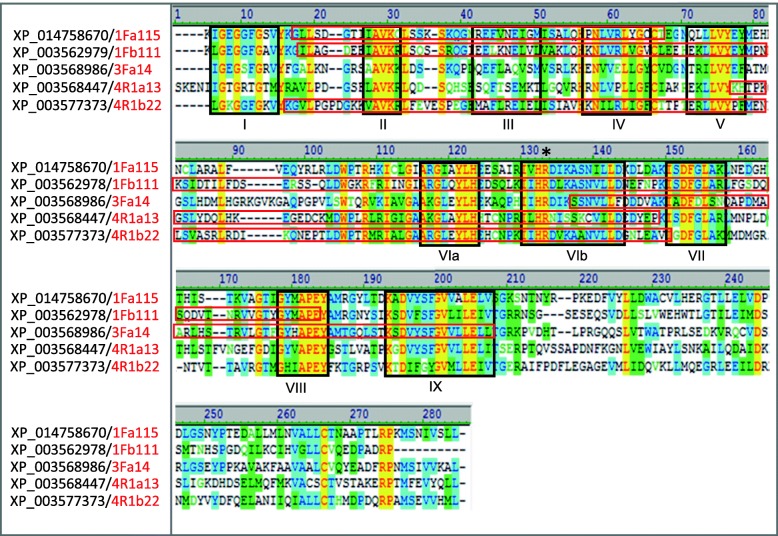
Alignment with ClustalW2 of the amino acid sequences of the serine/threonine tyrosine kinases of *Brachypodium* (black) with the highest similarity to those obtained in the PK-profiling (red). Black boxes indicate the 12 conserved subdomains of the protein kinases, with the exception of the subdomain X that are poorly conserved. Red boxes show the sequences cloned in this work. Asterisk points to the characteristic sequence of the active center (HRD). The colors of the amino acid refer to the degree of similarity: Black: Not similar; Yellow: Identical; Green: Similar; Blue: Conserved

The amplicons generated in PK-profiling were small, but the information provided by the cloned sequences was enough to classify them according to sequence similarity by BLAST searches. The clones were most similar to sequences of *B. distachyon*. This genome is the closest related to that of *Avena* that has been sequenced [46]. Translation and ClustalW2 alignment of each sequence cloned with the complete sequences of the most similar *B. distachyon* PKs allowed those of the MN841801–1 oat line to be deduced and characterized (Figs. 4 and 5). According to the domains they presented, they were classified into two groups: serine/threonine or mixed (serine/threonine-tyrosine). The characteristic amino acids motifs that define the 12 subdomains within the conserved catalytic domain of the PKs were clearly identified. As described by [47], subdomains I-IV of the amino-terminal region, the large carboxyl-terminal region with subdomains VI to XI, and the characteristic sequence of the active center in subdomain VIb, were all present.

The presence of PKs in the used SSH library similar to those obtained in the present work was explored by BLASTn, and some 67% of the sequences described were found in the library sequence pool. Three sequences (4R1a11, 3Fb11, and 4R1a13) were not represented in the SSH library. These match with kinase families likely under-represented in the transcriptome from which the library was generated. The next generation sequencing performed on the SSH library required a minimum number of readings of each sequence for accurate assignments to be made. However, PK-profiling allows identification and cloning of PK sequences, even if they are poorly represented in the cDNA examined. Further work should increase the number of fragments sequenced, but the present data indicate PK-profiling to be a good way of exploring and identifying specific sequences belonging to conserved PK families.

**Fig. 6:**
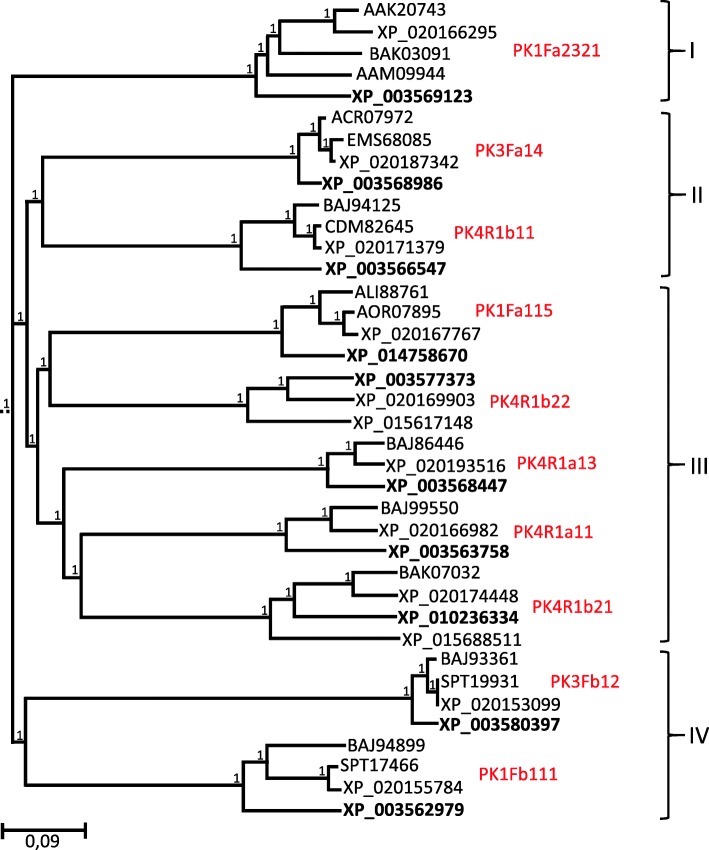
Phylogenetic tree of oat PKs and homologs sequences from close related species. The represented tree is the bootstrap consensus tree generated by the Neighbor-Joining method. A 100% of replicate trees showed the associate sequences clustered in each branch This is indicated by 1 next to the branches. Accessions numbers are described in Additional file 2. Oat PKs representing each cluster of sequences are indicated in red. Four well supported clades, I to IV were defined

The exact role of PKs in the defenses of oats against *P. coronata* is still to be determined. From studies in other plant species, it is known that phosphorylation is a major mechanism controlling protein activity in plant-pathogen interactions [7, 48, 49]. The phylogenetic tree of the oat PK cloned fragments and their most similar proteins of other cereals (Additional file 2) performed by the Neighbor-Joining method (Fig. 6) identified the putative function of the clones PK sequences. All the oat PK sequences analyzed have functions that have related to defense response. In the phylogenetic tree, four well-supported clades (100% bootstrap) were found. The largest clade, number III, contains sequences identified as LRR-RLK that are associated with innate immunity in plants. Thus, sequence 4R1b21 showed similarity to *Xa21* of rice that is identified as a pattern recognition receptor which confers broad-spectrum resistance against *Xhantomonas oryzae* pv. *Oryzae* [50, 51]. Cluster II contains sequences with serine/threonine kinase activity that did not contain receptor-domains and are cytoplasmic proteins but could act downstream the defense response. Such is the case of PK3Fa14 related to a *Pto*-interacting protein that mediates response defense to *Peudomonas syringae* in tomato [52]. Interestingly, cluster I contains sequences highly similar to the PK coded by the resistance *Lr10* gen of wheat [53]. The protein coded by this gene is a receptor-like kinase that functions in the abscisic acid (ABA) signaling pathway in response to biotic and abiotic stresses, in Arabidopsis. [54]. A putative role of ABA in partial resistance has been previously reported [28] and therefore sequence PK1Fa232 could act in this process. In addition, calcium-dependent PK included in cluster IV, like 3Fb12, have been described as participants in the early defense response to pathogens [55]. Members of this class of PKs are as Ca^2+^ sensors controlling transcriptional reprogramming of immune genes, mostly as positive regulators. However, calcium - dependent protein kinases have been also found as negative regulators in the first step of the resistance response [56], as we have detected for PK 3Fb12. It is downregulated in infected oat leaves at 48 hpi after infection (Fig. 3). The complexity of the plant immune response and the interplay among different actors would affect specially a proteins participating as mediators in signaling transductions. This would help to explain the different kinds of regulations observed for the PKs [55]. Less studied, the expression of cysteine-rich receptor kinases has been reported after induction with both biotic and abiotic agents [57]. Sequence 1Fb111, also included in cluster IV, is similar to this kind of protein. From this analysis, it could conclude that the majority of PKs analyzed here have putative functions at the first steps of the immune response. This is in agreement with results of our previous work [28] supporting the hypothesis that basal defense mechanisms are the main system operating in oat partial resistance to *P. coronata*.

## Conclusions

The present results show the subtraction step performed to obtain the SSH library used was appropriate, with a significant reduction achieved in the number of constitutive or downregulated sequences. A significant proportion of the amplified DNA sequences generated by the PK-profiling primers were similar to PK genes of related species. They were specifically expressed in the MN841801–1 line after their induction with *P. coronata*. PK-profiling would appear to be a useful tool for the identification of the PKs expressed in oats after challenge by *P. coronata* and perhaps by other pathogens.

## Additional files

Additional file 1: Primers sequence of clones analyzed by RT-qPCR. (DOCX 16 kb)

Additional file 2: Data used in the construction of the phylogenetic tree. Accession numbers of the protein kinases of *Brachypodium distachyon*, *Triticum tauschii*, *Triticum eaestivum*, *Hordeum vulgare*, *Avena sativa* and *Oryza brachyanta and O. sativa included in the phylogenetic tree that showed highest similarity to the clones analyzed. (DOCX 16 kb)

## Abbreviations

ABA: Abscisic acid
Arf: ADP-ribosylation factor
Avr: Gene Avirulence gene
cDNA: Complementary DNA
CRR: Cysteine-Rich repeats
Ct: Threshold point
GAPDH: GlycerAldehyde-3-Phosphate DeHydrogenase
hpi: Hours post infection
LRR: Leucine-Rich Repeats
MAPKs: Mitogen Activated Protein Kinases
MDH: Malate dehydrogenase
NBS: Nucleotide Binding Site
PK: Protein Kinase
QTLs: Quantitative Trait Loci
R gene: Resistance gene
RGAs: Resistant Gene Analogs
RLKs: Receptor Like Kinases
RNA-seq: RNA sequencing
RT-qPCR: Reverse Transcription-quantitative PCR
SAGA: Spt-Ada-Gcn5-Acetyl transferase
SSH: Suppression Subtractive hybridization
