# Supplementary material for: PK-profiling method for identifying the expression of resistance-associated genes in partially resistant oats to crown rust
Source: BMC Plant Biol. 2018 Dec 29;18:376. doi: 10.1186/s12870-018-1604-y (PMC6311036; doi:10.1186/s12870-018-1604-y)
Supplement: Supplementary file 1 — Primers sequence of clones analyzed by RT-qPCR. (DOCX 16 kb) [file 12870_2018_1604_MOESM1_ESM.docx]

| **Sequence name** | **Primers** |
| --- | --- |
| 4R1a11 | Fw: 5’-CTTTGCTCGAAAATCAGGGTCCA-3’  Rev: 5’-CCACTAGATTGCAAATTGCCATCG-3’ |
| 3Fa14 | Fw: 5´-TCAAACCAAGCTCCTGACATGGC-3’  Rev: 5’- TCCAGTCATTGCATACCTGATCCAA-3’ |
| 3Fb12 | Fw: 5’-TGCTCGCTAACAAGGACGACGA-3’  Rev: 5’- TGGTCCGTATTTCTTGCAGAGCAC-3’ |
| 4R1a13 | Fw: 5’-CGGTGAAGGATTCGAGGATTGC-3’  Rev: 5’-GCCCAAAGGCTCACTCTATGATCAG-3’ |
| 4R1b22 | Fw: 5’-TGACTGCTTCAAACTTCCCATCGA-3’  Rev: 5’-TGCTGCCCGTGGTTTGGAAT-3’ |
| 1Fb111 | Fw: 5’-TTCCTGGCGATGAAGAAATAGCAGT-3’  Rev: 5’-GTTCTTCCAAGCAAACACCGACAA-3’ |
| *1Fa115 | Fw: 5’-CAGCCATACAGCCTGACAAG-3’  Rev: 5’-CAGACAAGGGAACCGTGAAT-3’ |
| *4R1b21 | Fw: 5’-CCAGGCCCTTGAAATCAATACTGG-3  Rev: 5’-TGTCGCCGTGAAGGTGCTCA-3’ |

*qRT-PCR evaluated in Loarce et al. (2016)
