# Supplementary material for: PK-profiling method for identifying the expression of resistance-associated genes in partially resistant oats to crown rust
Source: BMC Plant Biol. 2018 Dec 29;18:376. doi: 10.1186/s12870-018-1604-y (PMC6311036; doi:10.1186/s12870-018-1604-y)
Supplement: Supplementary file 2 — Data used in the construction of the phylogenetic tree. Accession numbers of the protein kinases of Brachypodium distachyon, Triticum tauschii, Triticum eaestivum, Hordeum vulgare, Avena sativa and *Oryza brachyanta and O. sativa included in the phylogenetic tree that showed highest similarity to the clones analyzed. (DOCX 16 kb) [file 12870_2018_1604_MOESM2_ESM.docx]

| **Clone** | ***Brachypodium*** | ***T. tauschii*** | ***T. aestivum*** | ***Hordeum*** | ***Avena*** | ***Oryza*** |
| --- | --- | --- | --- | --- | --- | --- |
| **PK1Fa115** | XP_014758670 | XP_020167767 | AOR07895 | ALI88761 | ------- | ------- |
| **PK1Fa2321** | XP_003569123 | XP_020166295 | AAK20743 | BAK03091 | AAM09944 | ------- |
| **PK1Fb111** | XP_003562979 | XP_020155784 | SPT17466 | BAJ94899 | ------- | ------- |
| **PK3Fa14** | XP_003568986 | XP_020187342 | EMS68085 | ACR07972 | ------- | ------- |
| **PK3Fb12** | XP_003580397 | XP_020153099 | SPT19931 | BAJ93361 | ------- | ------- |
| **PK4R1a11** | XP_003563758 | XP_020166982 | ------- | BAJ99550 | ------- | ------- |
| **PK4R1a13** | XP_003568447 | XP_020193516 | ------- | BAJ86446 | ------- | ------- |
| **PK4R1b11** | XP_003566547 | XP_020171379 | CDM82645 | BAJ94125 | ------- | ------- |
| **PK4R1b21** | XP_010236334 | XP_020174448 | ------- | BAK07032 | ------- | XP_015688511 |
| **PK4R1b22** | XP_003577373 | XP_020169903 | ------- | ------- | ------- | XP_015617148 |
